# Predicting an opaque bubble layer during small-incision lenticule extraction surgery based on deep learning

**DOI:** 10.3389/fcell.2024.1487482

**Published:** 2024-10-30

**Authors:** Zeyu Zhu, Xiang Zhang, Qing Wang, Jian Xiong, Jingjing Xu, Kang Yu, Zheliang Guo, Shaoyang Xu, Mingyan Wang, Yifeng Yu

**Affiliations:** ^1^ Ophthalmic Center, The Second Affiliated Hospital, Jiangxi Medical College, Nanchang University, Nanchang, China; ^2^ Center of Ophthalmic, Heyou Hospital, Foshan, China; ^3^ School of Mathematics and Computer Sciences, Nanchang University, Nanchang, China; ^4^ Nanchang University, Nanchang, China

**Keywords:** deep learning, opaque bubble layer, small-incision lenticule extraction, artificial intelligence, complication

## Abstract

**Aim:**

This study aimed to predict the formation of OBL during femtosecond laser SMILE surgery by employing deep learning technology.

**Methods:**

This was a cross-sectional, retrospective study conducted at a university hospital. Surgical videos were randomly divided into a training (3,271 patches, 73.64%), validation (704 patches, 15.85%), and internal verification set (467 patches, 10.51%). An artificial intelligence (AI) model was developed using a SENet-based residual regression deep neural network. Model performance was assessed using the mean absolute error (*E*_*MA*_), Pearson’s correlation coefficient (*r*), and determination coefficient (*R* ^*2*^).

**Results:**

Four distinct types of deep neural network models were established. The modified deep residual neural network prediction model with channel attention built on the PyTorch framework demonstrated the best predictive performance. The predicted OBL area values correlated well with the Photoshop-based measurements (*E*_*MA*_ = 0.253, *r* = 0.831, *R* ^*2*^ = 0.676). The ResNet (*E*_*MA*_ = 0.259, *r* = 0.798, *R* ^*2*^ = 0.631) and Vgg19 models (*E*_*MA*_ = 0.31, *r* = 0.758, *R* ^*2*^ = 0.559) both displayed satisfactory predictive performance, while the U-net model (*E*_*MA*_ = 0.605, *r* = 0.331, *R* ^*2*^ = 0.171) performed poorest.

**Conclusion:**

We used a panoramic corneal image obtained before the SMILE laser scan to create a unique deep residual neural network prediction model to predict OBL formation during SMILE surgery. This model demonstrated exceptional predictive power, suggesting its clinical applicability across a broad field.

## Introduction

Small-incision lenticule extraction (SMILE) surgery is a widely employed and efficacious ocular surgery for treating myopia and astigmatism (Ang et al., 2021). Compared to keratorefractive surgery, SMILE has a higher level of predictability, precision, and security (Palme et al., 2022; Wu et al., 2014; Sekundo et al., 2011; Reinstein et al., 2014; Kobashi et al., 2017). A common intraoperative complications associated with SMILE is an opaque bubble layer (OBL). This is engendered by emission of pulsed light lasting 10^−15^ s at a wavelength of 1053 nm, which causes photofracture and the generation of bubbles (carbon dioxide + dihydrogen oxide) (Moshirfar et al., 2021; Mrochen et al., 2010). Subsequent bubbles coalescence creates an opaque area. An OBL causes cap lenticule adhesion, hampering dissection of lenticules. In severe cases, it may precipitate complications, such as epithelial breakthrough and cap perforation (Sahay et al., 2021; dos Santos et al., 2016; Son et al., 2017), which markedly affect the patient’s postoperative vision (Aristeidou et al., 2015). Hence, diminishing OBL formation during SMILE surgery holds promise for reducing complications and improving postoperative visual quality, with significant practical implications.

In recent years, artificial intelligence (AI) advanced ophthalmology significantly, presenting novel opportunities and challenges for ocular disease diagnosis, treatment, and management (Nuzzi et al., 2021). Through deep learning (DL) and machine learning, AI systems can swiftly and accurately detect lesions in fundus color photography, optical coherence tomography (OCT), and fundus fluorescein angiography (FFA), thereby assisting clinicians in early stage disease screening and diagnosis (Schmidt-Erfurth et al., 2018), and can predict the risk of glaucoma (Li et al., 2020; Xiong et al., 2022). Combined use of AI and patient data and surgical videos in cataract surgery holds significant promise for transforming surgical practice (Al Hajj et al., 2019).

We investigated the value of DL techniques for predicting OBL in SMILE surgery. Specifically, we attempted to predict formation of an OBL in femtosecond laser SMILE surgery by recognizing the panoramic view of the cornea before laser scanning and compared this prediction with the actual measured OBL area.

## Materials and Methods

### Research object

This cross-sectional retrospective study enrolled patients who underwent SMILE at the Ophthalmology Center of the second affiliated hospital of Nanchang University of between June 2021 and October 2022. All patients underwent SMILE surgery to treat myopia and astigmatism, by two experienced surgeons with 10 and 20 years’ refractive surgery experience in thousands of cases of photorefractive keratectomy, femtosecond-laser-assisted *in situ* keratomileusis (FS-LASIK), and SMILE surgeries.

This study follows the Declaration of Helsinki, is registered at ClinicalTrails.gov (identifier, NCT06577012), and approved by the ethics committee of the second affiliated hospital of Nanchang University (2024086).

Patients were included if they were aged ≥18 years, had preoperative spherical equivalent ≥−10.0 dioptre (D); corrected distance visual acuity ≥16/20; had a relatively stable refractive diopter (annual dioptre change in the past 2 years < 0.50 D); and had not worn contact lenses in the last 2 weeks. Patients were excluded if they had ocular diseases other than myopia and astigmatism, including keratoconus, severe corneal disease, severe dry eye, uncontrolled glaucoma, cataracts that seriously affected vision, or a history of ocular trauma; an ocular surgery history; and systemic diseases, such as psychiatric disorders, severe hyperthyroidism, systemic connective tissue diseases, or autoimmune diseases.

### Data collection

Data collection involved obtaining screenshots of the SMILE surgical video in a panoramic view of the intraoperative corneal and posterior lenticule cut scans. Additionally, the OBL area was measured in operated eyes (Son et al., 2017). Following completion of the side-cut during SMILE surgery, the video was immediately paused, and the image was captured in BMP format using the “screen capture” option. Subsequently, the BMP file was imported into Adobe Photoshop (PS) 2020 software (Adobe Systems, San Jose, CA), where the “Elliptical Marquee Tool” was utilised to select the total corneal area. The mean luminosity and standard deviation were recorded, and the percentage of pixels above the threshold (average brightness + two standard deviations) was documented as the OBL region (Son et al., 2017; Yang et al., 2023). Measurement of the OBL area was checked by a senior surgeon to eliminate overestimation or underestimation by the PS software. The actual measured OBL area are subsequently verified and recorded by senior surgeons.

### Data set building

All operations involved in building the data set were performed by professionally trained technicians.

#### Image preprocessing

**FIGURE 1:**
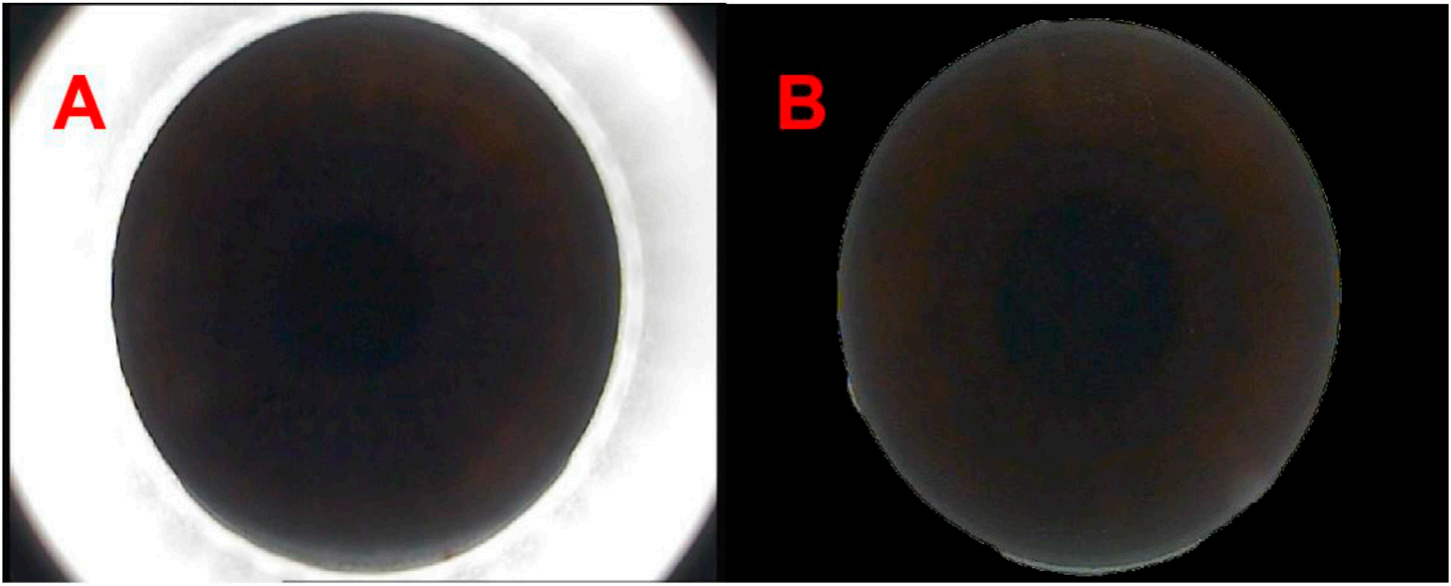
The panoramic view of corneal processed by Python [**(A, B)** respectively show the panoramas view of corneal captured from the VisuMax storage system (Carl Zeiss, Germany) and the panoramas view of corneal processed by Python].

Considering the surgical instruments in the images captured from the VisuMax storage system (Carl Zeiss, Oberkochen, Germany), our initial approach involved the use of OpenCV within Python (https://pypi.org/project/opencv-python/) to remove instruments around the cornea. Subsequently, a binary process was employed to separate the cornea from the instrument, with the corneal regions individually labelled through a maximum connected region analysis. The corneal size was calculated using the ellipse-fitting method. Upon determining the corneal dimensions, a mask image was generated, and the image bit operation was executed using the cv2.bitwise_and () functions to yield a complete corneal image, effectively zeroing all neighbouring pixels within the image (Figure 1).

#### Data partitioning

**FIGURE 2:**
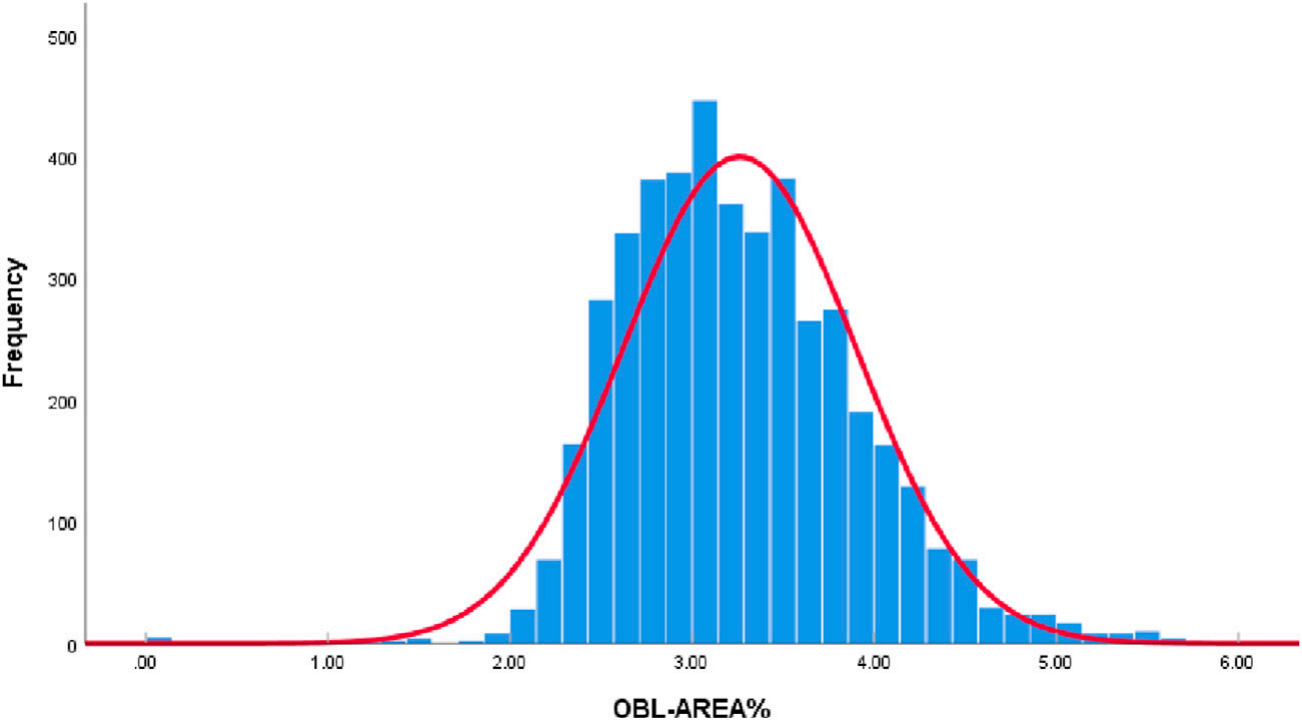
The histogram data of OBL areas.

All collected OBL area data were plotted as a histogram. According to the Kolmogorov-Smirnov test, the data did not conform to a normal distribution (*P* < 0.05). However, given the large sample size of this study (4,442 eyes) and the “Bell Curve” characteristics exhibited by the histogram data of OBL areas, which approximate a normal distribution (Figure 2), it is acceptable to consider the data as normally distributed (Vetter, 2017; Muntaner et al., 1996).

**FIGURE 3:**
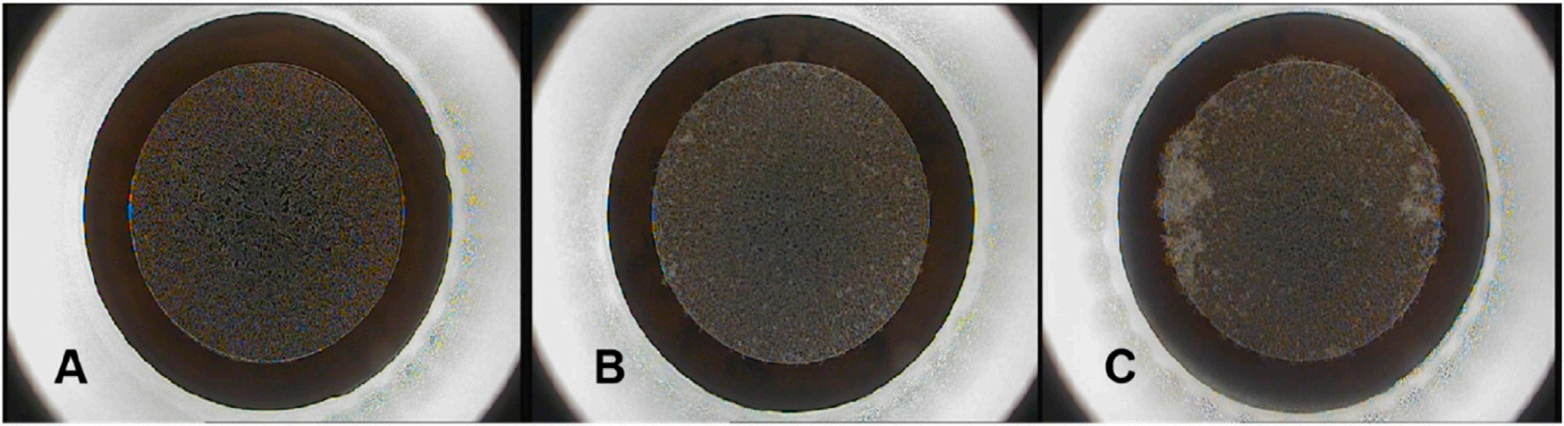
OBL area measured in operated eye. [**(A–C)** respectively show the OBL generated during SMILE surgery captured by the VisuMax storage system. After processing by PS software and checking by senior surgeons, 3A represents the OBL area <3%, 3B represents the OBL area 3%–4%, and 3C represents the OBL area >4%].

Based on the proportion of the OBL area in the panoramic view of the cornea, we divided the data into three categories: <3%, 3%–4%, and >4% (Figure 3), consisting of 1,619, 2,126, and 697 items, respectively. Given that most of the OBL area data fell within the range of 3%–4%, random partitioning was performed to allocate this subset into the training set (60%), verification set (20%), and test set (20%). The remaining data were divided into proportions of 80%, 10%, and 10%.

The training set comprised 60% of the OBL data falling within the range of 3%–4% and 80% of the data outside this range (<3% and >4%), totalling 3,271 patches. These datasets were used to train the DL network to predict the OBL percentage area.

The verification set included 20% of the OBL data within and 10% of the data outside the 3%–4% range, amounting to 704 patches. These patches were used in real-time monitoring of the network training progress and the adjustment of network hyperparameters.

For the test set, we used 20% of the OBL data that fell within and 10% of the data that fell outside the 3%–4% range, resulting in a selection of 467 patches. By verifying the prediction outcomes of the test set, key evaluation indicators, such as the mean absolute error (*E*_*MA*_), Pearson correlation coefficient (*r*), and determination coefficient (*R* ^*2*^), were calculated (Table 1).

**TABLE 1:** Specific compositions of training set, verification set and test set.

Categories	The number of patches			
	<3%	3% ∼ 4%	>4%	Total
Training set	1,296	1,418	557	3,271
Verification set	162	472	70	704
Test set	161	236	70	467

### Residual regression deep neural network based on SENet

Predicting the area of the OBL formed during SMILE based on an RGB image of the eye can be considered as a regression problem. Therefore, we designed a deep neural network incorporating the Sque-and-Excitation Networks (SENet) and ResNet architecture to solve this regression prediction task.

SENet is an influential network architecture (Hu et al., 2020) that introduces a pioneering feature-relabeling strategy called the attention mechanism. This approach obtains significant weights for each channel through a learning process. Subsequently, these discerned weights are utilised to enhance the crucial features relevant to the given task, while the impact of less essential features is simultaneously suppressed.

Moreover, residual neural networks devised by He et al. (2016) and his team at Microsoft Research tackled the issue of “degeneration” by introducing a groundbreaking concept known as the “shortcut” or “skip connection” mechanism. These connections resolved challenges encountered when training a neural network with excessive depth.

In the present study, the SENet module was integrated into a deep residual network. To streamline the number of parameters in the model, we have chosen to employ the same global average pooling (GAP) as the original creators to process the features extracted from the convolutional layer. Simultaneously, the loss function was refined to the mean square error (MSE) to enhance the precision of the loss calculation in the regression prediction task.

#### SENet module

**FIGURE 4:**
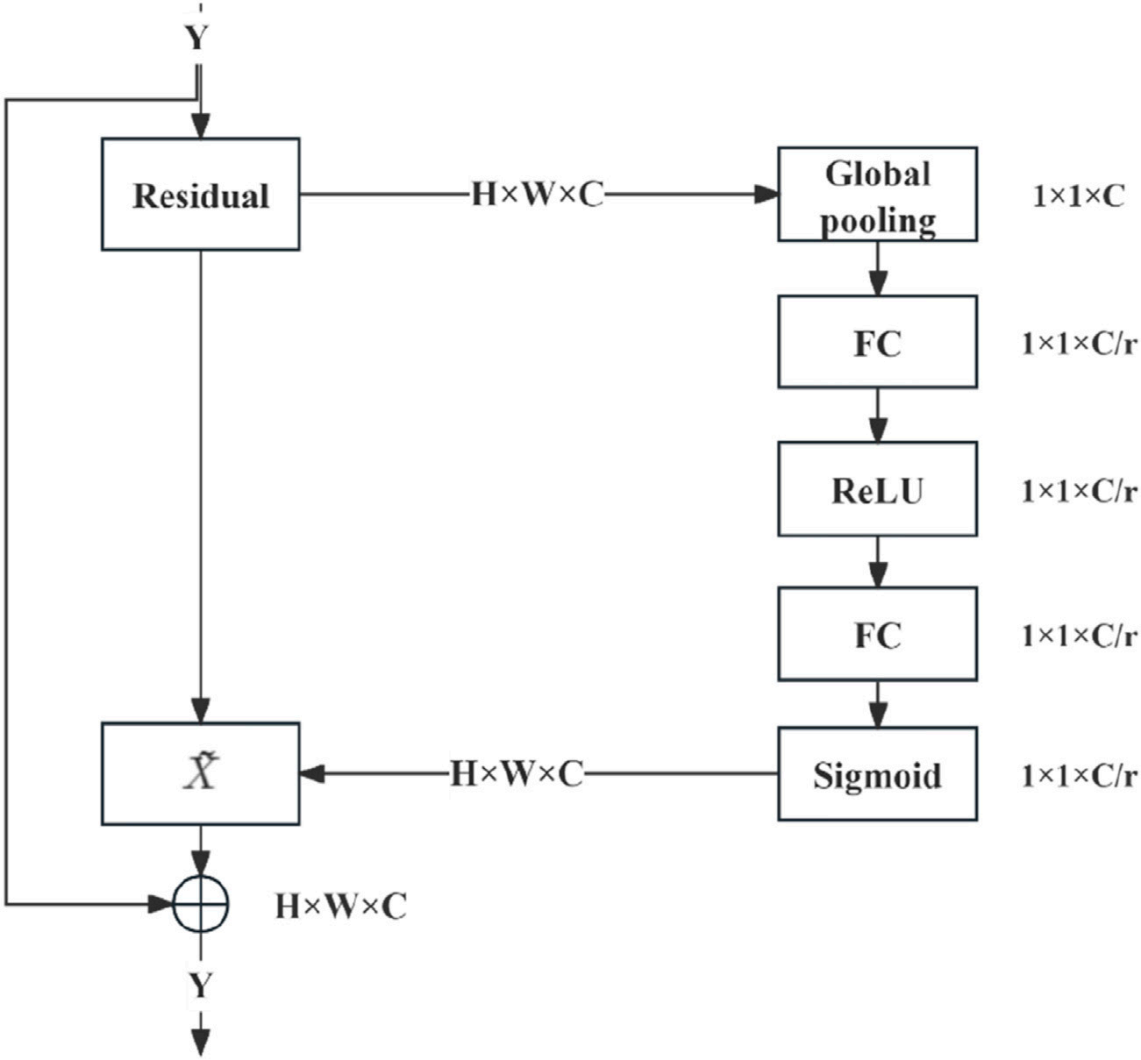
Combination of SENet model and deep residual network module.

The combination of SENet and the deep residual network module (Figure 4) entails executing weighted operations on the original image at the conclusion of each residual block, including the GAP, fully connected layer, ReLU operation, and sigmoid function. These operations calculate the weight values for each channel, which are then leveraged for multiplication. SENet was combined with the deep residual network by integrating it into the residual module following the last block in the module.

#### Residual network

**FIGURE 5:**
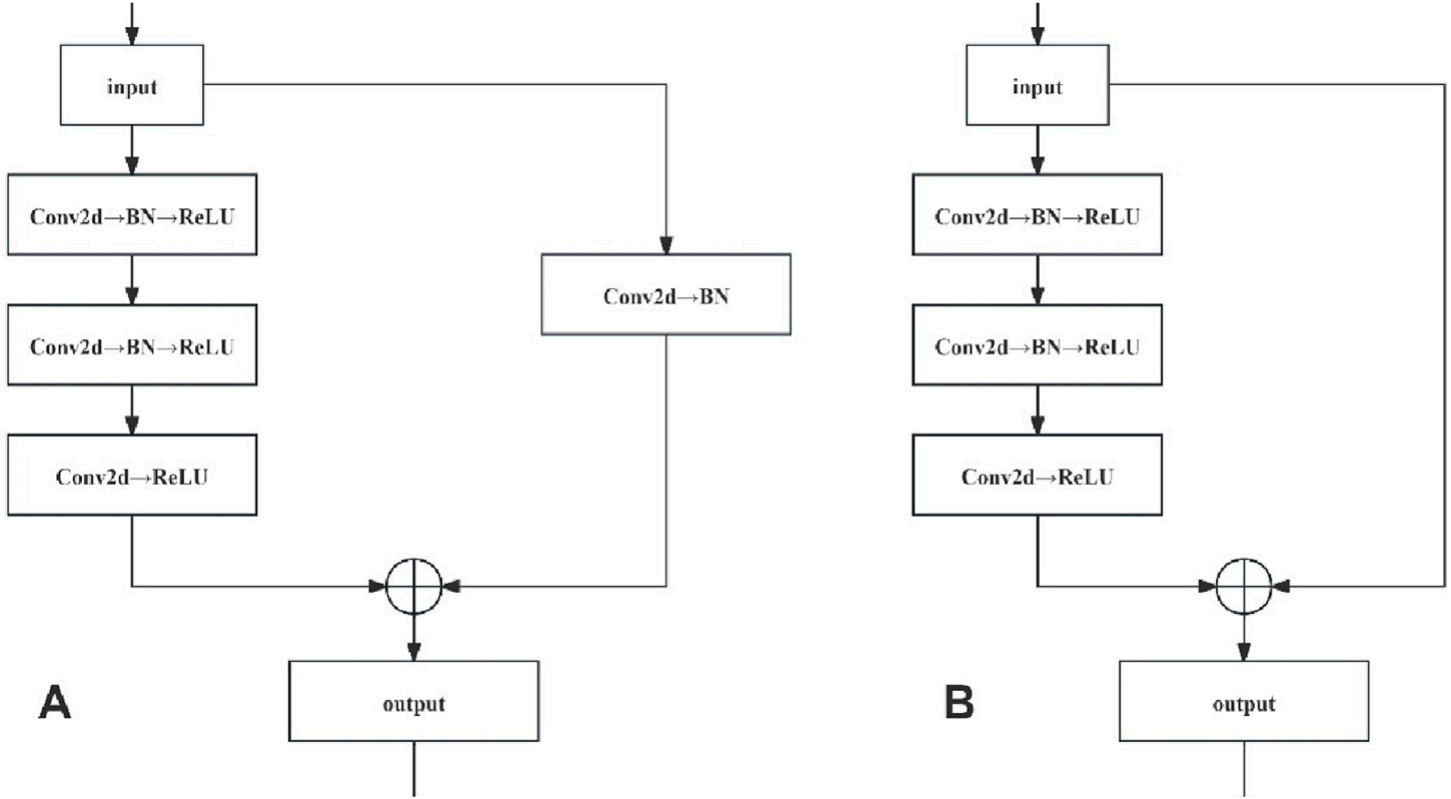
**(A, B)** shows the network structure of Conv Block and Identity Block respectively.

Residual networks were constructed by stacking the convolutional (Conv) and identity blocks. As the input and output dimensions of the Conv blocks differed, they were used to adjust the dimensions of the network. Conversely, the identity blocks maintained uniform input and output dimensions, thus augmenting the network depth. The network structures of the Conv and identity blocks are shown in Figure 5.

The network structure of the Conv locks is characterised by a bifurcated structure, and the input data are divided into two branches, with the first branch undergoing a three-layer convolution operation to obtain a feature map, while the second branch undergoes a single-layer convolution operation to obtain another feature map. These two are added, and the result is subjected to a ReLU operation (i.e., assuming that a negative value is 0). Finally, the ReLU operation results are taken as the Conv block module output. In the network structure of the identity block, only one branch of input data exists. After three convolution operations without changing the dimensions, which are directly added (i.e., residual connection), a ReLU operation is performed on the result of the sum, which is finally used as the output of the identity block module.

#### Loss functions and optimizer

In this study, the MSE loss *EMS (MSE)* was chosen as the loss function (Equation 1), calculated as follows:

$$E_{MS}\left(y,y^{\prime }\right)=\frac{1}{n}\sum_{i=1}^{n}{\left(y_{i}-y_{i}^{\prime }\right)}^{2}$$ (1)

Stochastic Gradient Descent is an optimisation algorithm commonly used for large-scale datasets and DL model training. It calculates the gradient of the loss function by selecting one of the samples and verifies that the direction of the negative gradient updates the weights to reduce the value of the loss function. The loss function, which is the optimal value of the weights, is minimised by iteratively updating the weights.

#### Evaluation indexes

In this study, *E*_*MA*_, Pearson’s correlation coefficient *r*, and the determination coefficient *R* ^*2*^ were chosen as the evaluation indices, calculated as follows (Equations 2–4):

$$E_{MA}=\frac{1}{n}\sum_{i=1}^{n}\left\|y_{i}-y_{i}^{\prime }\right\|$$ (2)

$$r=\frac{\sum_{i=1}^{n}\left(y_{i}^{\prime }-\bar{y}\right)\left(y_{i}^{\prime }-{\bar{y}}^{\prime }\right)}{\sqrt{\sum_{i=1}^{n}{\left(y_{i}-\bar{y}\right)}^{2}\sum_{i=1}^{n}{\left(y_{i}^{\prime }-{\bar{y}}^{\prime }\right)}^{2}}}$$ (3)

$$R^{2}=\frac{\sum_{i=1}^{n}{\left(y_{i}^{\prime }-\bar{y}\right)}^{2}}{\sum_{i=1}^{n}{\left(y_{i}-\bar{y}\right)}^{2}}$$ (4)

Where $\bar{y}\text{ and }\bar{y}ˊ$ are the means of the true and predicted values of the sample, respectively.

$$\bar{y}=\frac{1}{n}\sum_{i=1}^{n}y_{i},\bar{yˊ}=\frac{1}{n}\sum_{i=1}^{n}y_{i}ˊ$$

### Experimental environment

The DL regression model used in this study was based on implementation under the PyTorch framework, running on a server equipped with two NVIDIA 3090 GPUs. The version of Pytorch was 1.10.0, the version of CUDA was 11.1, and the operating system was Ubuntu 18.04. Each GPU had 128 GB of RAM and 24 GB of graphics memory.

The original image was normalised and the pixel values were scaled to the range [0, 1]. A modified deep residual neural network with channel attention was used for training, and a dropout function was introduced in the fully connected layer, to reduce overfitting. During the training process, the batch size was set to 48, the iteration epoch was set to 200, the learning rate of the stochastic gradient descent was set to 10–4, and every 50 iterations reduced the learning rate to 1/10th of the initial value. During model training, the evaluation metric, Pearson’s correlation coefficient *r*, was recorded for the validation set, and the model with the largest *r* value was selected. Finally, models were evaluated using the test set for each correlation metric, to verify their predictive performance.

### Surgical procedure

Each patient received 0.3% gatifloxacin eye gel for 3 days before surgery as a prophylactic measure against infection. Immediately before surgery, the nurse administered 0.5% procaine hydrochloride eye drops in a series of one drop every 5 min, followed by two consecutive drops to provide surface anaesthesia to the affected eye.

A VisuMax femtosecond laser system (Carl Zeiss), with a 135-nJ pulse energy of and 4.5-μm spot spacing was used. The corneal cap thickness was set at 100–120 μm, with a 7.5-mm diameter. The femtosecond laser scanning sequence included the following steps: posterior lenticule, lenticule side-cut, anterior lenticule, and cap side-cut. The cap side-cut was set at 2 mm (at the 12 o’clock position, at a 90° angle). The transition zone for astigmatism treatment was 0.1 mm (Table 2). The surgeon controlled the eyeball by using microtoothed forceps in the left hand and femtosecond lens separation in the right hand to dissect the anterior plane of the lenticule, followed by posterior plane dissection and lenticule extraction.

**TABLE 2:** SMILE surgical parameters.

Surgical parameters	Range
Wavelength/nm	1,053
Pulse duration/fs	400
Pulse emission frequency/kHz	500
Optical zone/mm	6.5
The transition zone for astigmatism treatment/mm	0.1
Cap diameter/mm	7.5
Cap thickness/μm	100–120
The line and spot separations/μm	4.5
Cap side cut angle/°	90
Incision width/mm	2
Energy/nJ	135

All patients began using the following medications on the first day after surgery: 0.3% gatifloxacin eye gel (four times/day, instilled in the eye for 1 week), 0.1% flumilone eye drops (four times/day, instilled in the eye for 4 weeks, decreased once a week), and sodium hyaluronate eye drops (four times/day, instilled in the eye for 4 weeks).

### Statistical methods

IBM SPSS Statistics for Windows version 26.0 (IBM Corp., Armonk, NY, USA) was used for statistical processing of the general information. Microsoft Excel 2018 (Microsoft Org., Redmond, WA) was used to organise and tabulate the data, and GraphPad Prism 9.0 (GraphPad Inc., La Jolla, CA) was used for data analysis. All measurements are expressed as mean ± standard deviation ( $\bar{x}\pm s$ ). Count data are expressed as *n* (%).

In this study, *E*_MA_, *r* and *R* ^*2*^ were used as the model evaluation indices. *E*_MA_ was affected by the size of the processed data; therefore, the *E*_MA_ only reflected the mean absolute error value, which was not readable. In Pearson’s correlation test, *r* = 0.8–1.0 was defined as a very strong correlation, *r* = 0.6–0.8 as a strong correlation, *r* = 0.4–0.6 as a moderate correlation, *r* = 0.2–0.4 as a weak correlation, and *r* = 0.0–0.2 as a poor correlation or lack of correlation. *R* ^*2*^ was used to reflect the goodness-of-fit of the model and ranged from 0 to 1. *R* ^*2*^ > 0.4 indicated reliable goodness-of-fit.

## Results

### General information and DL model prediction performance

This study included 2,265 patients (4,442 eyes) with a mean age of 21.88 ± 5.32 years. Of these, 68.12% were male patients, and the mean measured OBL area was 3.26% ± 0.64%.

The proposed algorithm was validated using a test set. It was also compared with classical network architectures, such as U-net, Vgg19, and ResNet50, to evaluate its predictive performance (Table 3). Method in this study and the ResNet50 model have been described in detail in the METHODS section. The U-net network comprises four layers, with the image undergoing subsampling and upsampling four times, respectively. The convolution kernel sizes for each layer are 64, 128, 256, and 512, respectively. Each subsampling process is connected with max pooling, and the upsampling process utilizes deconvolution and the ReLU activation function of the U-Net network. Additionally, the VGG19 model consists of 19 layers in total, including 16 convolutional layers and 3 fully connected layers. The convolutional part is divided into five blocks, with the respective block counts being 2, 2, 4, 4, and 4. Each block utilized max pooling for connectivity, and then connect three fully connected layers, and finally output the result through the softmax activation function.

**TABLE 3:** Evaluation indexes of different DL models in the test set.

Deep neural networks	E_MA_	MSE	R^2^	r
U-net	0.605	0.375	0.171	0.331
VGG19	0.31	0.185	0.559	0.758
ResNet50	0.259	0.154	0.631	0.798
Method in this study	0.253	0.136	0.676	0.831

According to Table 3, ResNet50 exhibits a smaller *E*_MA_, and larger *R* ^*2*^ and *r* than did U-net and Vgg19. Our proposed method showed some improvements over ResNet: it slightly reduced the *E*_MA_ and increases the *R* ^*2*^ and *r* values. However, omitting GAP from the model resulted in a significant reduction in the size of the model (MB). Taken together, the method proposed in this study performed the best in terms of predictive effectiveness.

### Visual analysis of model predictions

**FIGURE 6:**
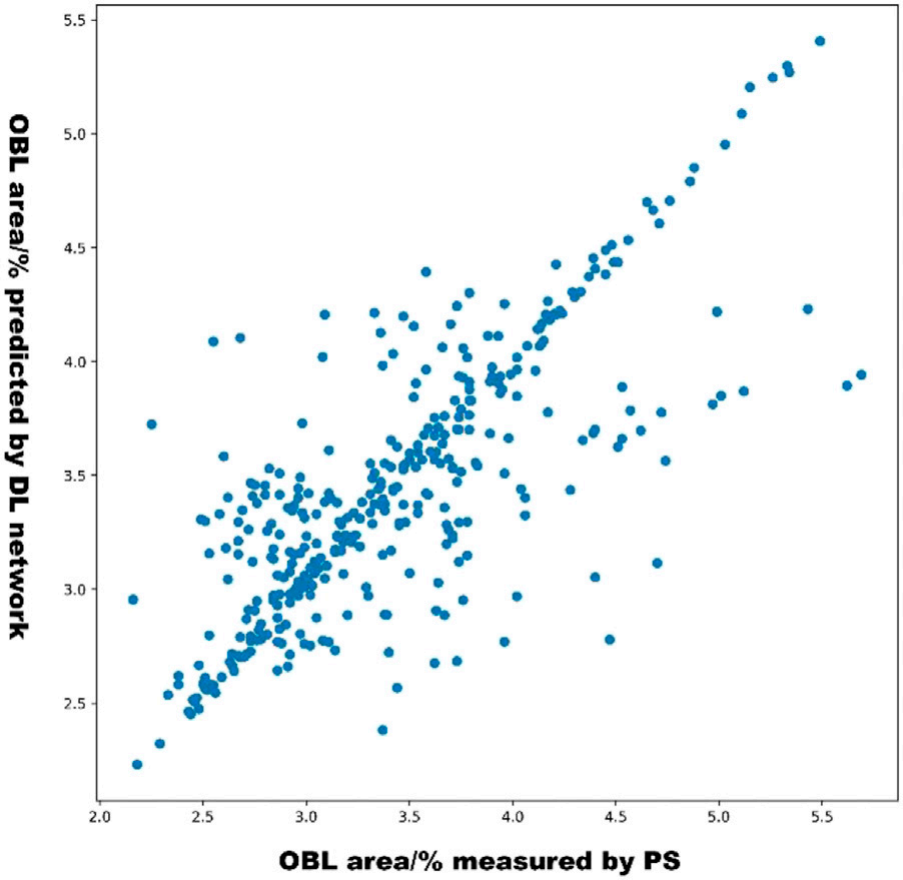
Distribution of OBL measured values and predicted values.

The scatterplot in Figure 6 shows the distribution of the OBL predictions of our model versus the OBL measurements. These data correlated well (*r* = 0.831).

**FIGURE 7:**
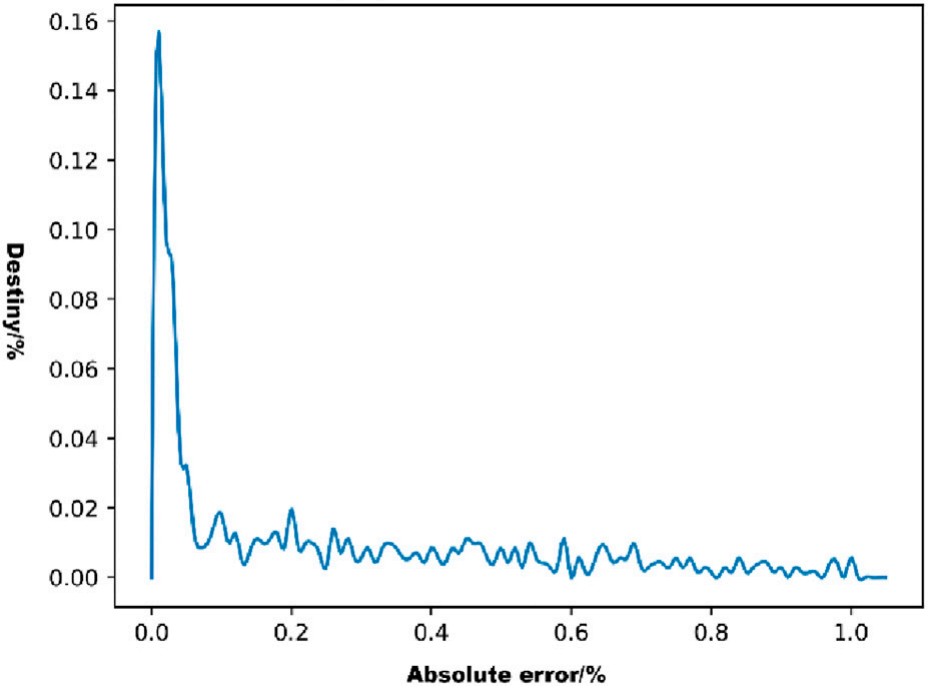
Density distribution of absolute errors.

The distribution of the absolute errors in the model predictions was observed using a density line graph of the absolute errors (Figure 7). As shown in Figure 7, most of the absolute error values were distributed below 0.2%, which represents a small error range.

## Discussion

In this study, we trained and evaluated four DL models to predict the OBL area during surgery, based on a panoramic corneal image obtained before conducting SMILE laser scanning. To confirm the best-performing DL model, we used an internal validation set and designed a deep neural network model incorporating channel attention and a residual module (*E*_MA_ = 0.253, *r* = 0.831, and *R* ^*2*^ = 0.676). The model-matching effect and predictive performance were good. The DL model designed in this article could assist surgeons in predicting potential OBL areas in patients preoperatively, enabling the adjustment of surgical parameters to mitigate OBL formation, such as reducing the laser energy, replacing the suction ring or modifying the thickness of the corneal cap (Yang et al., 2023; Wu et al., 2020). This adjustment is crucial for reducing adverse impacts on surgical outcomes and postoperative visual recovery, bearing significant practical implications.

An OBL is an intraoperative complication of SMILE. Its formation is mainly related to the excessive accumulation of interlaminar corneal gas generated during photorupture of the corneal stroma by the femtosecond laser, particularly when a posterior lenticule is scanned. In previous studies, Liu et al. (2014), Courtin et al. (2015), He et al. (2022), and others have investigated the risk factors for OBL in FS-LASIK and have reported that higher myopia, greater central corneal thickness, and larger corneal diameter were associated with OBL formation. Ma et al. (2018) and Son et al. (2017) suggested that the central corneal thickness and residual stromal thickness are significant independent risk factors for OBL formation, with a higher likelihood of OBL occurrence in eyes in which a thinner lenticule is created during the SMILE procedure.

To date, AI has demonstrated significant potential in the screening, diagnosis, progression prediction, and supportive treatment of ocular diseases (Xiong et al., 2022; Wan et al., 2023; Lin et al., 2019). The widespread application of AI technology is expected to have a revolutionary impact on ophthalmology, but AI algorithms have not yet been used to predict OBL formation during SMILE, as we have done here. The methodology integrated various disciplines, such as the physical characteristics of femtosecond lasers, OBL formation mechanisms, computer image processing technology, and DL algorithms. The AI prediction process was achieved by extracting effective feature parameters from a panoramic corneal image scanned by the femtosecond laser. This study provided a detailed theoretical explanation of the algorithm implementation process and experimental results, and demonstrated that our AI prediction model has high accuracy and superior performance compared with traditional multiple linear regression models.

This study had some limitations. The DL model developed in this study focused specifically on predicting OBL formation during SMILE but did not predict the specific region or quadrant of OBL occurrence and the weight of the forming factors that influence OBL. Considering the complexity and variety of SMILE complications in clinical practice, creating an AI model that can predict all intra-and postoperative complications would entail a lengthy accumulation process. The study’s training and test data were relatively limited, highlighting the need for more extensive test sample data collection and the establishment of a robust data platform to test system adequacy. Additionally, the reliance on data from only one hospital for the test, training, and verification sets in this study introduces a certain level of inaccuracy. Future research should involve gathering data from diverse regions and hospitals for further testing and refinement.

## Conclusion

In this study, a DL network, including a residual convolutional structure of residual modules, was constructed to extract image features, output regression predicted values by the fully connected layer, and channel attention. The model performance was verified on real datasets and exhibited a higher statistical correlation and a lower *E*_*MA*_ than classical network architecture models.

## Data Availability

The raw data supporting the conclusions of this article will be made available by the authors, without undue reservation.

## Abbreviations

OBL, Opaque bubble layer; SMILE, Small incision lenticule extraction; AI, Artificial intelligence; DL, Deep learning; E_MA_, Mean absolute error; r, Pearson’s correlation coefficient; R^2^, Determination coefficient; PS, Adobe Photoshop; D, Dioptre.
